# *In vivo *activity of *Sapindus saponaria *against azole-susceptible and -resistant human vaginal *Candida *species

**DOI:** 10.1186/1472-6882-11-35

**Published:** 2011-05-04

**Authors:** Edílson Damke, Joyce K Tsuzuki, Diógenes AG Cortez, Izabel CP Ferreira, Thâmara A Bertoni, Márcia R Batista, Lucélia Donati, Terezinha IE Svidzinski, Márcia EL Consolaro

**Affiliations:** 1 Department of Clinical Analysis and Biomedicine, State University of Maringá, Paraná, Brazil; 2 Department of Pharmacy and Pharmacology, State University of Maringá, Paraná, Brazil; 3 Department of Cell Biology, Federal University of Paraná, Curitiba, Paraná, Brazil

**Keywords:** Sapindus saponaria, vaginal yeasts, antifungal activity, in vivo

## Abstract

**Background:**

Study of *in vivo *antifungal activity of the hydroalcoholic extract (HE) and n-BuOH extract (BUTE) of *Sapindus saponaria *against azole-susceptible and -resistant human vaginal *Candida *spp.

**Methods:**

The *in vitro *antifungal activity of HE, BUTE, fluconazole (FLU), and itraconazole (ITRA) was determined by the broth microdilution method. We obtained values of minimal inhibitory concentration (MIC) and minimum fungicide concentration (MFC) for 46 strains of *C. albicans *and 10 of *C. glabrata *isolated from patients with vulvovaginal candidiasis (VVC). VVC was induced in hyperestrogenic Wistar rats with azole-susceptible *C. albicans *(SCA), azole-resistant *C. albicans *(RCA), and azole-resistant *C. glabrata *(RCG). The rats were treated intravaginally with 0.1 mL of HE or BUTE at concentrations of 1%, 2.5% and 5%; 100 μg/mL of FLU (treatment positive control); or distilled water (negative control) at 1, 24, and 48 h after induction of the infection, and the progress of VVC was monitored by culturing and scanning electron microscopy (SEM). The toxicity was evaluated in cervical cells of the HeLa cell line.

**Results:**

The extracts showed *in vitro *inhibitory and fungicidal activity against all the isolates, and the MIC and MFC values for the *C. glabrata *isolates were slightly higher. *In vivo*, the SCA, RCA, and RCG infections were eliminated by 21 days post-infection, with up to 5% HE and BUTE, comparable to the activity of FLU. No cytotoxic action was observed for either extract.

**Conclusions:**

Our results demonstrated that HE and BUTE from *S. saponaria *show inhibitory and fungicidal activity *in vitro*, in addition to *in vivo *activity against azole-resistant vaginal isolates of *C. glabrata *and azole-susceptible and resistant isolates of *C. albicans*. Also considering the lack of cytotoxicity and the low concentrations of the extracts necessary to eliminate the infection *in vivo*, HE and BUTE show promise for continued studies with purified antifungal substances in VVC yeast isolates.

## Background

Natural products have been traditionally used in the control of various diseases, because they are a source of many active compounds that show multiple therapeutic effects, in addition to constituting models for the synthesis of a large number of pharmaceuticals [1]. The fruit of *Sapindus saponaria *L. (Sapindaceae), a medium-sized tropical tree found principally in America and India, has shown antimicrobial activity [2-4], but few studies have been carried out with this plant.

In a recent study, members of our research group isolated and identified the principal constituents of the n-BuOH extract (BUTE) of the pericarps of *S. saponaria*: two acetylated triterpene saponins, S1 and S2, and also an acyclic oligoglycoside. The same group also demonstrated excellent inhibitory action *in vitro *of the water-alcohol extract (HE) and BUTE against the yeasts *Candida albicans *and non- *C. albicans *isolated from patients with vulvovaginal candidiasis (VVC)[5], signaling the possibility of using this plant as an antifungal agent in this pathology. In spite of these recent investigations of the constituents and biological properties of *S. saponaria*, few *in vivo *studies have yet been carried out to establish a correlation with the *in vitro *results.

VVC is caused by abnormal growth of these yeast-like fungi in the mucosa of the female genital system [6]. It affects millions of women annually and can cause great discomfort, affecting sexual and affective relations and job performance, and is considered an important problem of world public health [7]. Management of patients with VVC is often difficult because of the few available therapeutic options; and furthermore, cross-resistance of vaginal *C. albicans *has been detected to itraconazole and fluconazole, which are the antifungal agents of choice for treatment of this pathology [7,8]. In view of the need for new therapeutic options for VVC and the promising *in vitro *inhibitory activity of *S. saponaria *L. against yeasts, we conducted a study of antifungal activity *in vivo *of HE and BUTE against azole-susceptible and -resistant human vaginal *Candida *spp., and also of the cell toxicity of these extracts.

## Methods

### Plant and components

Dry pericarps of the fruits of *S. saponaria *were collected on the campus of the State University of Maringá, Paraná, Brazil (UEM). The plant was identified by staff members of the UEM Department of Botany, and an exsiccate was deposited in the Herbarium of this institution (HUM 11710). The dried pericarps of the fruits (450.0 g) of *S. saponaria *were ground and extracted with EtOH:H_2_O (9:1) at room temperature, by a process of dynamic maceration with constant mechanical stirring. The extraction was carried out in an amber flask, maintained at ambient temperature, for six consecutive days, for 6 h per day. The extract was concentrated under low pressure in a rotary evaporator, at a temperature of 40°C. After elimination of the solvent, the extract was frozen in liquid nitrogen and lyophilized in a Martin Christ Alpha 1-2 freeze dryer. The lyophilized extract was stored in a closed plastic flask and kept frozen.

The HE of the pericarp (50.15 g) was chromatographed in a column (ϕ_i _= 4.0 cm) of silica gel 60 (Merck, Darmstadt, Germany), and eluted with solvents of increasing polarity including hexane, dichloromethane, ethyl acetate, and methanol (Merck, Darmstadt, Germany). The solvents were evaporated at a temperature of 40°C, frozen in liquid nitrogen, and lyophilized in a Martin Christ Alpha 1-2 freeze dryer. The lyophilized dichloromethane, hexane, ethyl acetate, and methanol fractions were stored in closed containers and kept frozen. The methanol extract was suspended in H_2_O and extracted with n-butanol, which after evaporation gave a solid residue (28.9 g) (BUTE), which was also lyophilized. The structures were established with the use of spectroscopic methods (^1^H and ^13^C NMR, HSQC, HMBC, and ESI/MS) and by comparing them with literature data [2,4].

### Yeast isolates

For the experiments on *in vitro *susceptibility, 56 vaginal isolates obtained from patients with VVC were tested, including 46 isolates of *C. albicans *and 10 of *C. glabrata*, which are part of a bank of yeasts at the Medical Mycology Laboratory/UEM. In this yeast bank, aliquots of the yeasts are stored after their identification in 10% glycerinated water at -20°C. The yeasts used in this study were isolated and identified in 2008, by classical methods [9-11] and also by rDNA sequencing [12]. Prior to each experiment, the isolates were reactivated in Sabouraud Dextrose Broth (SDB) (Difco, Detroit, USA) at 25°C for 24/48 h, seeded on Sabouraud Dextrose Agar (SDA) (Difco, Detroit, USA) with chloramphenicol (2.0 mg/mL), and incubated again under the previous conditions. A new subculture was made in CHROMágar Candida^® ^(Probac, France) to assure the purity of the isolates.

### Antifungal agents

Stock solutions of fluconazole (FLU) (5000 mg/mL; Pfizer Inc., NY, USA) and itraconazole (ITRA) (1000 mg/mL; Janssen Pharmaceutica, Titusville, NJ, USA) were prepared. From the first prepared solution, new stock solutions of FLU and ITRA were prepared at a concentration of 10 times that of the final test concentration, and diluted in bicarbonate-free RPMI-1640 with L-glutamine, supplemented with 2% dextrose and buffered to pH 7.0 with 0.165 M morpholinopropanesulfonic acid (MOPS) (Sigma, Steinheim, Germany). The lyophilized HE and BUTE extracts were dissolved in sterile distilled water to obtain a 10 mg/mL solution of each extract.

### Determination of minimum inhibitory (MIC) and fungicidal concentrations (MFC)

The tests of susceptibility of *Candida *spp. to FLU and ITRA were carried out according to the broth microdilution method recommended by the CLSI (Clinical Laboratory Standards Institute, 2002), and of susceptibility to HE and BUTE according to the same document, with adaptations for natural products [13,14].

A suspension of yeast compatible with 1.0 to 5.0 × 10^6 ^colony-forming units per mL (CFU/mL) was prepared in sterile saline, adjusting the cell density by means of a spectrophotometer (Spectronic 70, Bausch & Lomb, USA) at 530 nm with 90 ± 2% transmittance. From this suspension, new dilutions were made: 1:50 in sterile saline, and then 1:20 in RPMI (Sigma, Steinheim, Germany), thus obtaining the desired final inoculum of 0.5 to 2.5 × 10^3 ^CFU/mL. The tests were carried out in sterilized plastic microplates (TPP Zellkultur Test Plate 96F, Switzerland) containing 96 wells arranged in 8 rows labeled A to H, each row with 12 wells numbered 1 to 12. Each row (A-H) corresponded to one isolate, and each well received 100 μL of the measured inoculum, except for the 12th well which was the negative control. Aliquots of 100 μL of RPMI (Gibco, NY, USA) were distributed from columns 2 to 11. Aliquots of 100 μL of FLU, ITRA, HE, or BUTE, prepared as previously described, were added to the columns of the microplates, and from column 2 a 2-fold serial dilution was made up to the 10th well (diluitions between 0.125 and 64.0 μg/mL for FLU, 0.03 and 16.0 μg/mL for ITRA, and 9.0 and 5000.0 μg/mL for HE and BUTE).

For each isolate tested, negative controls (only RPMI) and positive (RPMI plus inoculum, with no antifungals added) were included, for growth and for the possible action of the diluent of the extracts or drugs (only butanol, ethanol, or polyethyleneglycol 400 with the inoculum). On each microplate a strain of *Candida parapsilosis *(ATCC 22019) was included as the reference yeast. The plates thus set up were incubated in an oven at 35°C with daily monitoring. After 48 h they were read for FLU and ITRA in a microplate reader (Asys Hitech GmbH, Eugendorf, Austria) and after 72 h for the extracts, by visual comparison of the reflection in a mirror.

The MIC for FLU/ITRA was determined as the lowest concentration of the drug that was capable of inhibiting 50% of the growth of each yeast, with reference to its respective positive control [13]. The criteria for definition of susceptibility/resistance to FLU/ITRA were those established by CSLI [13]. For HE and BUTE, the MIC was considered to be the smallest concentration of the extract that was capable of inhibiting 100% of the inoculum compared to its respective positive control [14]. The MIC_50 _and MIC_90 _for the drugs and extracts were defined as the MICs capable of inhibiting 50% and 90% of the isolates, respectively [13].

To determine the MFC, subcultures from all wells showing growth inhibition were made by seeding 5.0 μL in SDA at 25°C. After 48 h, the CFU were counted to determine the viability. All the assays for determination of the MIC and MFC were carried out in duplicate, independently. The MFC for HE and BUTE was considered as the lowest concentration that impeded the growth of 100% of the inoculum. The MFC_50 _and MFC_90 _for the extracts were defined as the MFCs capable of inhibiting 50% and 90% of the isolates, respectively.

### Experimental vaginal infection

A rat model was used as previously described [15], with some adaptations. The experiments were carried out with three yeast isolates, which were selected according to the results of the *in vitro *tests: *C. albicans *susceptible to FLU and ITRA (SCA), *C. albicans *resistant to ITRA (RCA), and *C. glabrata *resistant to both antifungals (RCG).

The experiments were carried out with groups of five rats for each isolate, in duplicate and on two different days. Non-oophorectomized Wistar rats (*Rattus norvegicus*) weighing from 200 to 300 g and 70 days old (from the UEM Central Animal Facility) were used. The rats received subcutaneous injections of estradiol valerate (Sigma, Steinheim, Germany) at a concentration of 0.2 mg/week/rat. Six days after the first injection of the hormone, the animals were inoculated intravaginally with 10^8 ^yeast cells/mL of each isolate tested, prepared in 0.1 mL of sterile saline and counted in a Neubauer chamber.

For the treatment, HE and BUTE were administered intravaginally (0.1 mL at 1%, 2.5%, and 5.0% in distilled water) at 1, 24, and 48 h after the induction of the vaginal infection. Rats receiving FLU (3 doses of 100 μg intravaginally over the same time periods as the extracts) or distilled water served as positive and negative treatment controls, respectively. The kinetics of the *Candida *vaginal infection in the treated and control rats was monitored in each animal by means of the number of CFU/mL in the vaginal fluid at 24 and 48 h after induction of the infection, and on days 5, 7, 14, and 21. The animal experimentation carried out in this investigation was approved by the UEM Committee on Ethical Conduct in Animal Use (Protocol No. 013/2006, Opinion No. 050/2006).

### Toxicity in cervical cells

Cells from the HeLa human cervical line were previously cultured in Eagle's minimum essential medium (MEM, PPA Laboratories, Germany) supplemented with 10% fetal bovine serum (FBS, Laborclin, Brazil), 0.1 mM non-essential amino acids, and 1 mM sodium pyruvate, at 37°C in a humid oven with 5% CO_2_. In the exponential growth stage, the cells were diluted in the same medium and plated in volumes of 0.2 mL of a suspension of 2.5 × 10^5 ^cells per well in a 24-well plate (Corning Glass, New York, USA), and incubated in the same conditions overnight to allow them to form a cell monolayer. The culture medium was replaced by serial dilutions of the HE or BUTE extracts at 1%, 2.5%, 5%, and 10% concentrations, in triplicate. The control wells contained only cells and culture medium. The microplate was incubated again at 37 °C for 24 h, and the extracts were replaced by a trypsin-EDTA solution to undo cell adhesion, followed by addition of 0.2 ml of PBS with 50% trypan blue. Live and dead cells in each well were counted with the aid of light microscopy.

### Scanning electron microscopy (SEM)

The vaginal epithelium of rats infected with CAS, CGR, and CAR before and after treatment with HE and BUTE was observed by SEM. After 48 h of infection and also at the end of treatment, the rats to be analyzed by SEM were killed with an overdose of anesthetics (Ketamine and Xylazine, Parke-Davis Co, Morris Plains, NJ, USA). The vagina was removed, washed, fixed in a solution of 2.5% glutaraldehyde in 0.1 M cacodylate buffer (Sigma Chemical, St. Louis, MO, USA), and dehydrated in an ascending ethanol series. The critical point was obtained in a Balzers CPD-010 (Balzers Instruments, Balzers, Liechtenstein) with carbonic gas. Metallization in gold was carried out in a Balzers SCD-030 (Balzers Instruments, Balzers, Liechtenstein). The vagina, uterine cervix, and tissue sections of all rats were observed and photographed with a JEOL-JSM 6360 LV scanning electron microscope (JEOL Ltd, Tokyo, Japan) at the Electron Microscopy Center, Federal University of Paraná/Curitiba/Brazil.

### Statistical analysis

The results were analyzed using Student's *t *test and Tukey's test for multiple comparisons of the different *in vivo *experimental treatment situations. The significance level was set at 5%. The tests were carried out by means of the program Graph Pad Prismâ version 3.0 (Graph Pad Software Inc.).

## Results

### Plants and components

The presence of two acetylated triterpene saponins was confirmed: saponin S1, hederagenin-3-O- (3,4-di-O-acetyl-β-D-xylopyranosyl)- (1→3)-α-L-ramnopyranosyl- (1→2)-α-L-arabinopyranoside; and saponin S2, hederagenin-3-O-(4-O-acetyl-β-D-xylopyranosyl)-(1→3)-α-L-ramnopyranosyl-(1→2)-α-L-rabinopyranoside; and also an acyclic oligoglycoside-1 (OGSA-1) in HE and BUTE, as previously described [5].

### Determination of *in vitro *minimum inhibitory and fungicidal concentrations

The majority of the *C. albicans *isolates proved to be susceptible *in vitro *to FLU and ITRA simultaneously (n = 38) and a few were resistant to ITRA (n = 3), but there was no resistance to FLU. Some of the *C. glabrata *strains proved to be resistant to FLU (n = 2) and ITRA (n = 4) and to both antifungals simultaneously (n = 2) (Table 1). HE and BUTE *in vitro *inhibited all the yeast isolates tested, including those that were susceptible-dose dependent (SDD) to FLU and resistant to FLU and/or ITRA. The values of MIC_50 _and MIC_90 _were higher for FLU, ITRA, HE, and BUTE in the isolates of *C. glabrata*. However, the variation of the MIC for HE and BUTE was the same for *C. albicans *(9.0-≤5.0 × 10^3 ^μg/ml) and very similar for *C. glabrata *(75.0-≤5.0 × 10^3 ^μg/ml and 36.0-≤5.0 × 10^3 ^μg/ml, respectively) (Table 1).

**Table 1:** Anti-*Candida *activity *in vitro *of the hydroalcoholic (HE) and butanolic (BUTE) extracts of *Sapindus saponaria *in comparison to fluconazole (FLU) and itraconazole (ITRA)

	*Drugs*	*C. albicans (46)^b^*	*C. glabrata (10)^b^*
^a^MIC range	FLU	0.125-32 (43)^c^, (3)^d^, (0)^e^	2.0-64.0 (5)^c^, (4)^d^, (1)^e^
	ITRA	0.03-1.0 (38)^c^, (5)^d^, (3)^e^	0.125-8 (3)^c^, (3)^d^, (4)^e^
	HE	9.0-≤5.0 × 10^3^	36.0-≤5.0 × 10^3^
	BUTE	9.0-≤5.0 × 10^3^	75.0-≤5.0 × 10^3^
MIC_50_	FLU	0.25	8.0
	ITRA	0.03	0.25
	HE	9.0	36.0
	BUTE	36.0	75.0
MIC_90_	FLU	16.0	64.0
	ITRA	0.5	8.0
	HE	310.0	620.0
	BUTE	310.0	5.0 × 10^3^

^f^MFC range	HE	9.0-≤5.0 × 10^3^	36.0-≤5.0 × 10^3^
	BUTE	9.0-≤5.0 × 10^3^	75.0-≤5.0 × 10^3^
MFC_50_	HE	310.0	2.5 × 10^3^
	BUTE	150.0	2.5 × 10^3^
MFC_90_	HE	310.0	5.0 × 10^3^
	BUTE	310.0	5.0 × 10^3^

^a ^Minimum inhibitory concentration - MIC (μg/mL); MIC_50 _and MIC_90 _for drugs and extracts: MIC capable of inhibiting 50% and 90% of the isolates, respectively; In parentheses: the number of isolates tested (b); the number of sensitive isolates (c); the number of susceptible-dose-dependent isolates (d); and the number of resistant isolates (e); Sensitive isolates: MIC ≤ 8.0 μg/mL for fluconazole (FLU) and ≤ 0.125 μg/mL for itraconazole (ITRA); susceptible-dose-dependent isolates: MIC between 16.0 and 32.0 μg/ml for FLU, and 0.25 and 0.5 μg/ml for ITRA; resistant isolates: MIC ≥ 64.0 μg/ml for FLU and ≥ 1.0 μg/ml for ITRA. ^f ^Minimum fungicidal concentration - MFC; The MFC_50 _and MFC_90 _are the MFCs capable of inhibiting 50% and 90% of the isolates, respectively.

Both extracts also exhibited fungicidal activity (MFC) *in vitro *for all the yeasts tested, independently of whether they were susceptible or resistant to azoles. There was little variation among MFC_50 _and MFC_90 _(Table 1). The MFC values were similar to the range of the MIC, confirming the important *in vitro *antifungal activity.

### Experimental vaginal infection

**Figure 1:**
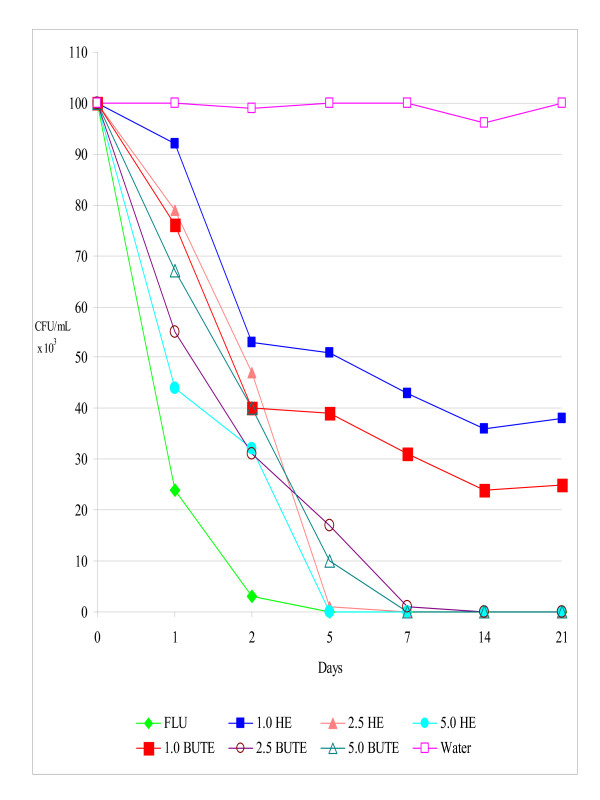
**Colony-forming units (CFU/mL) of yeasts in the vaginal exudate of Wistar rats treated intravaginally with hydroalcoholic (HE) and butanolic (BUTE) extracts of *Sapindus saponaria *at 1.0%, 2.5%, and 5.0%, 100 μg of fluconazole, or sterile distilled water at 1, 24, and 48 h after induction of the vaginal infection, followed for up to 21 days**. Each curve represents the mean (± standard deviation) of the CFU of five rats, in two independent experiments. Experimental vaginal infection by *Candida albicans *susceptible to itraconazole and fluconazole (SCA).

**Figure 2:**
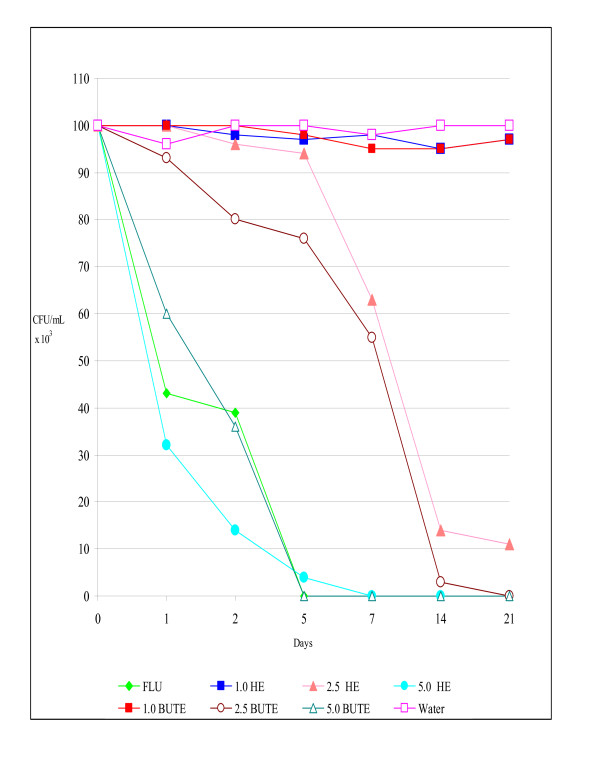
**Colony-forming units (CFU/mL) of yeasts in the vaginal exudate of Wistar rats treated intravaginally with hydroalcoholic (HE) and butanolic (BUTE) extracts of *Sapindus saponaria *at 1.0%, 2.5%, and 5.0%, 100 μg of fluconazole, or sterile distilled water at 1, 24, and 48 h after induction of the vaginal infection, followed for up to 21 days**. Each curve represents the mean (± standard deviation) of the CFU of five rats, in two independent experiments. Experimental vaginal infection by *C. albicans *resistant to itraconazole (RCA).

**Figure 3:**
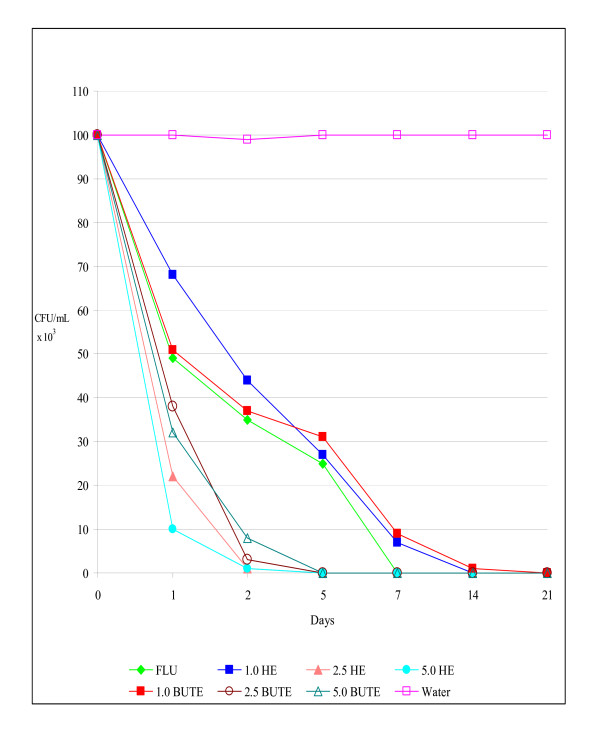
**Colony-forming units (CFU/mL) of yeasts in the vaginal exudate of Wistar rats treated intravaginally with hydroalcoholic (HE) and butanolic (BUTE) extracts of Sapindus saponaria at 1.0%, 2.5%, and 5.0%, 100 μg of fluconazole, or sterile distilled water at 1, 24, and 48 h after induction of the vaginal infection, followed for up to 21 days**. Each curve represents the mean (± standard deviation) of the CFU of five rats, in two independent experiments. Experimental vaginal infection by *C. glabrata *resistant to itraconazole and fluconazole (RCG).

The results *in vivo *confirmed those *in vitro*, since the infection by SCA, RCA, and RCG was eliminated by 21 days post-infection, with a maximum concentration of 5% of HE and BUTE. In these experiments, the untreated control rats remained infected until the end of all the assays (CFU/mL between 96 and 1.0 × 10^3^) (Figures 1, 2, 3).

In the infection by SCA, the inhibitory activity of FLU was superior only to that of HE and BUTE at 1% (p < 0.001), which at this concentration showed similar behavior (p > 0.05) and inhibited fungal growth compared to the negative control, but did not lead to elimination of the infection. The treatments with 2.5% and 5.0% HE and BUTE showed similar profiles of inhibition of infection, comparable to FLU (p > 0.05) (Figure 1).

In the RCA infection, FLU showed better inhibitory activity than 1% and 2.5% HE (p < 0.001) and 1% BUTE (p < 0.001). The HE at 1% and 2.5%, and 1.0% BUTE did not eliminate the infection, showing similar behavior to that of the untreated control (p > 0.05). For HE, the 5% concentration showed the best inhibitory activity (p < 0.001), as did the 2.5% and 5.0% concentrations of BUTE. There was no difference in the action between these last two concentrations, and also between the HE and BUTE at the 5% concentration (p > 0.05) (Figure 2). The RCA was resistant *in vitro *only to ITRA, and the positive control of the treatment was carried out with FLU for all yeasts tested, which although it is also an azole, showed excellent activity *in vivo*. No treatment was carried out with ITRA itself, because no vaginal formulations of this antifungal exist.

For RCG, the inhibitory activity of HE and BUTE at all concentrations tested was excellent and similar to that of FLU (p> 0.05). At the 1% concentration of both extracts, there was a significant decrease in the CFU count in the first days of infection (p < 0.05), and the infection was eliminated on day 14 in the experiment for HE, and on day 21 for BUTE. At 2.5% and 5.0%, both extracts showed the same activity, with elimination of the infection on day 5 (p > 0.05); while the FLU treatment eliminated the infection on day 7 (Figure 3). In general, HE in a concentration of 5% and BUTE in concentrations of 2.5% and 5% were capable of eliminating the infection induced by the different yeasts tested.

### Toxicity in cervical cells

**Figure 4:**
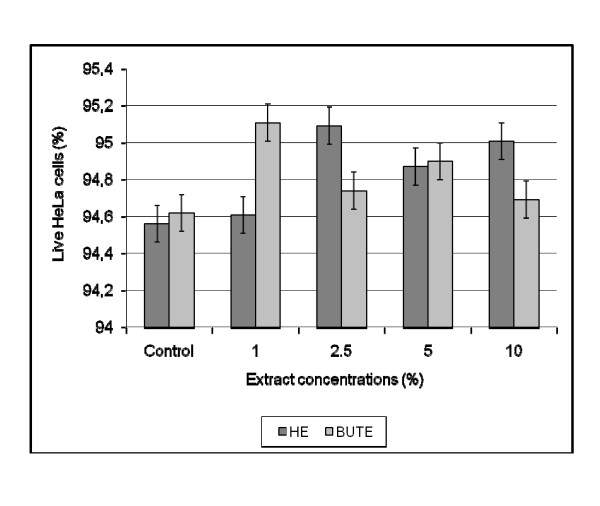
**Percentage of live HeLa cells after contact with different concentrations of the hydroalcoholic (HE) and butanolic (BUTE) extracts from *S. saponaria*, with no significant difference between the controls and extracts at all concentrations tested (p > 0.05)**. Mean of live cells in the concentrations of HE = 94.22 ± 0.1555; Mean of live cells in the concentrations of BUTE = 94.41 ± 0.1131 (IC 95% = -0.6295 to 0.2575).

The percentage of live HeLa cells did not vary between the controls and tests, and between HeLa cells exposed to different concentrations of HE and BUTE (p > 0.05). The mean of live cells in the different HE concentrations was 94.22 ± 0.1555, and in the BUTE concentrations was 94.41 ± 0.1131 (IC 95% = -0.6295 to 0.2575) (Figure 4).

### SEM

**Figure 5:**
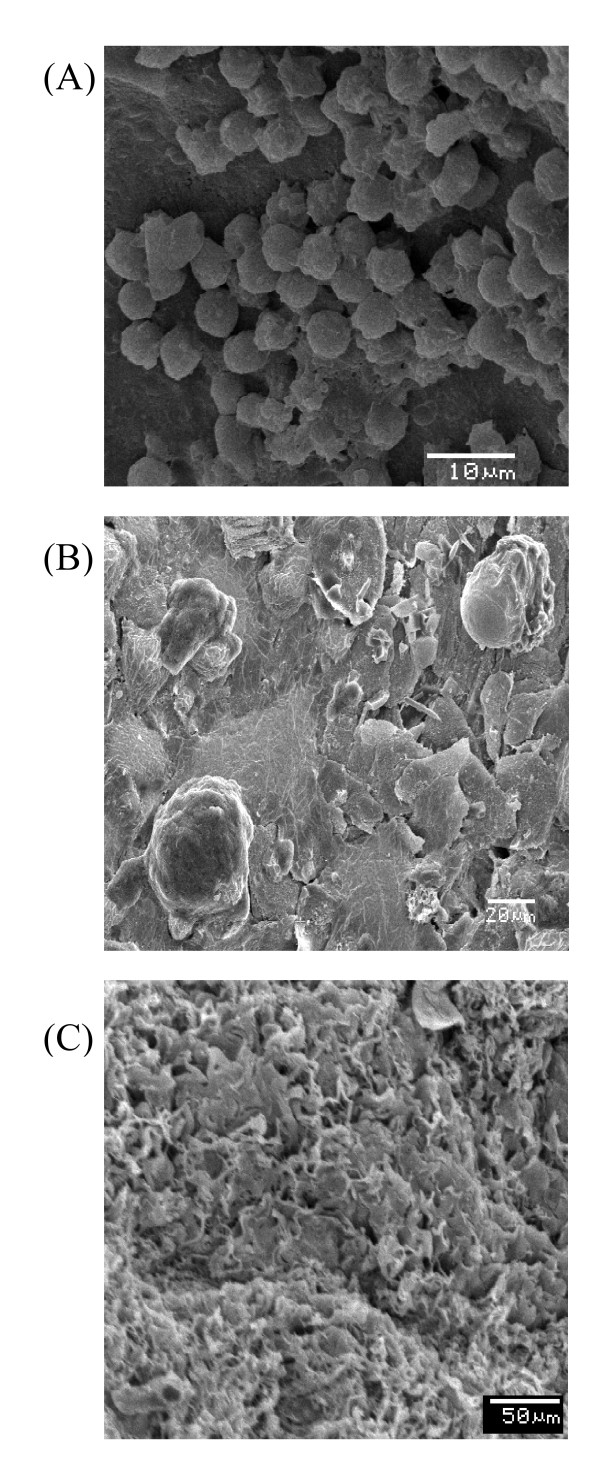
**MEV of the vaginal epithelium of hyperestrogenic Wistar rats after infection with *Candida albicans***. In (A) many yeasts adhered to the epithelium. In (B) larger detail of the adhesion of *C. albicans *to the anucleate cells of the vaginal epithelium, characteristic for the state of pseudo-estrus. In (C) epithelium composed only of anucleate scales, without yeasts, after treatment with fluconazole and with the extracts from *S. saponaria*.

The morphology of the vaginal epithelium of the hyperestrogenic rats infected by SCA, RCA, and RCG was indistinguishable upon examination by SEM images. As a result of this, we selected figures of RCA, which showed a profile of elimination of infection with slightly higher concentrations of the extracts than for SCA and RCG. Figure 5 shows several yeast cells of *C. albicans *adhered to the epithelium (A); greater detail of the adhesion of *C. albicans *to the anucleate cells of the vaginal epithelium, characteristic of the state of pseudo-estrus (B); and epithelium constituted only by anucleate cells, without yeasts (C), before and after treatment with FLU or with the extracts of *S. saponaria*. Thus, the SEM confirmed the results of the yeast cultures, with respect to the development of the experimental infection as well as its post-treatment elimination in the conditions tested.

## Discussion

Phytochemical analyses of some species of the genus *Sapindus *have shown that they are rich in triterpenoid saponins, containing oleanoic acid and hederagenin with aglycones [4]. The presence of these compounds was also confirmed in *S. saponaria*. In general, saponins have shown antifungal activity against *C. glabrata, C. albicans, Trichosporon beigeli, Penicillum avelaneum, Pyriculata oryzae, Cryptococcus neoformans, Coccidioidis immitis*, and *Saccharomyces cerevisiae*, as well as against the dermatophytes *Microsporum canis *and *Trichophyton mentagrophytes *[16-18]. Hederagenin isolated from the pericarps of *Sapindus mukurossi *exhibits potent antifungal activity against *Epidermophyton floccosum, Trichophyton mentagrophytes, T. rubrum, Sabouradites canis*, and *C. albicans*[18].

The constituents previously identified in the HE and BUTE in *S. saponaria *and confirmed by us, that is, S1, S2, and OGASA-01, are very possibly the same substances that are responsible for its antifungal action. Because of their antimicrobial activities, the saponins have been the target of many studies for the purpose of developing phytotherapeutic options for treatment of infections, that are possibly less toxic, more efficaceous, and economically accessible [19-21]. According to Francis et al. [22] the principal mechanism for the antifungal activity of the saponins is their interaction with steroids of the fungal membrane. This same study mentioned plants containing saponins with proven antifungal activity, among them *Kalopanax pinctus *against *C. albicans *and *Cryptococcus neoformans*, and *Aspargus officinalis *against different types of fungi.

The *in vitro *susceptibility tests of the FLU, ITRA, HE, and BUTE against vaginal yeasts were conducted with the goal of screening isolates for *in vivo *activity, to enable comparisons between the activities *in vitro *and *in vivo *and of the degree of antifungal activity for extracts. With respect to the *in vitro *susceptibility test for antifungal azoles, a few *C. abicans *were resistant to ITRA, but not to FLU, in concordance with other studies that also recently demonstrated resistance to azoles among vaginal isolates of this yeast [9,14]. Some isolates of *C. glabrata *were resistant to ITRA and FLU and also to both antifungals simultaneously, also in concordance with studies that demonstrated that vaginal isolates of non- *C. albicans*, principally *C. glabrata*, are less susceptible to azoles than is *C. albicans *[9,23].

Duarte et al. [24] proposed a classification for the inhibitory activity of plant extracts based on MIC values, so that MICs below 500 μg/mL represent strong inhibition, MICs between 600 and 1500 μg/mL moderate inhibition, and MICs above 1600 μg/mL weak inhibition. According to this classification and from the values of MIC_50 _and MIC_90 _obtained for the isolates of *C. albicans*, HE and BUTE demonstrated strong inhibitory activity, and moderate to strong activity against *C. glabrata *(Table 1). Tsuzuki et al. [5] have also demonstrated *in vitro *inhibitory and fungicidal activities of extracts of *S. saponaria *against some vaginal isolates of *C. albicans *and non- *C. albicans*. However, the degree of inhibitory activity was not determined.

In the *in vivo *tests, FLU and HE in a concentration of 5% and BUTE in concentrations of 2.5% and 5% were capable of eliminating the infection induced by the different yeasts tested (SCA, RCA, and RCG), including those that were resistant to *in vitro *tests. These results for *in vitro *resistant *C. albicans *and principally *C. glabrata *are important because there are few treatment options available for management of patients with VVC caused by these resistant yeasts [9,14]. The polyene derivatives nystatin and amphotericin B are the only presently available fungicidal drugs. The use of these medications is limited, principally because of their toxicity, and some isolates with dose-dependent susceptibility or resistance to these antifungals have been found [9,14]. Notably, FLU, the antifungal that is most frequently used to treat VVC, is only fungistatic. Therefore, even though the fungicidal activity (MFC) of the extracts of *S. saponaria *is not exceptional, the inhibitory activity (MIC) appears to us to be promising. However, our experiments did not use a previously purified antifungal substance, which would be necessary for more definite conclusions about antifungal activity.

Furthermore, it must be considered that *C. glabrata *is the second most frequently isolated species in cases of VVC, preceded only by *C. albicans*; and that in some human populations the rate of isolation of non- *C. albicans *yeasts has increased [25,26], emphasizing the importance of the antifungal activity of *S. saponaria *and of continuing studies with this plant.

In general, HE in a concentration of 5% and BUTE in concentrations of 2.5% and 5% were capable of eliminating the infection induced by the different yeasts tested. The results also evidenced the importance of correct identification of the yeasts in cases of VVC, as well as the determination of their *in vitro *profile of susceptibility to commercially available antifungals, because there were clear differences among the different isolates in the susceptibility profile, both *in vitro *and *in vivo*.

The results for cell toxicity indicate an absence of toxicity of the extracts to the cervical cells, a positive sign for the continuity of studies with *S. saponaria*. However, the animal toxicity is yet to be determined. Jacobs [27] has previously demonstrated the absence of cellular toxicity of *S. saponaria*. Interestingly, in tumor cells the toxicity appears to change, since Quetin-Leclerq et al. [28] demonstrated cytotoxic activity of saponins isolated from certain plant species, among them *S. mukorossi*, in B16 melanoma cells and HeLa human tumor cells, and Meyer et al. [29] demonstrated cytotoxic activity of the ethanol extract of *S. saponaria *on cells from an ascitic tumor.

## Conclusions

Our results demonstrated that HE and BUTE from *S. saponaria *show inhibitory and fungicidal activity *in vitro*, in addition to *in vivo *activity against azole-resistant vaginal isolates of *C. glabrata *and azole-susceptible and resistant isolates of *C. albicans*. Also considering the absence of cytotoxicity and the low concentrations of the extracts necessary to eliminate the infection *in vivo*, HE and BUTE constitute a promising source to continue studies with purified antifungal substance in VVC yeast isolates. There is still a need to determine the mechanisms of antifungal activity in order to validate the use of *S. saponaria *as an antifungal phytotherapeutic product.

## Abbreviations

HE: hydroalcoholic extracts of *Sapindus saponaria*; BUTE: n-BuOH extract of *Sapindus saponaria*; FLU: fluconazole; ITRA: itraconazole; MIC: minimal inhibitory concentration; MFC: minimum fungicide concentration; VVC: vulvovaginal candidiasis; SCA: azole-susceptible *C. albicans*; RCA: azole-resistant *C. albicans*; RCG: azole-resistant *C. glabrata*; UEM: State University of Maringá, Paraná, Brazil; SDB: Sabouraud Dextrose Broth; SDA: Sabouraud Dextrose Agar; CFU/mL: colony-forming units per mL; CLSI: Clinical Laboratory Standards Institute; SEM: Scanning electron microscopy

## Competing interests

The authors declare that they have no competing interests.

## Authors' contributions

ED conceived the study, participated in its design and coordination, and helped to draft the manuscript. JKT prepared the extracts. DAGC and ICPF analyzed the plant components and helped to draft the manuscript. TAB carried out the *in vitro *susceptibility tests. MRB performed the statistical analyses and helped to draft the manuscript. LD carried out the scanning electron microscopy and helped to draft the manuscript. TIES conceived the study, participated in its design and coordination, and helped to draft the manuscript. MELC carried out the cell toxicity analyses, participated in the study coordination, and helped to draft the manuscript. All authors read and approved the final manuscript.

## Pre-publication history

The pre-publication history for this paper can be accessed here:

http://www.biomedcentral.com/1472-6882/11/35/prepub
